# The chaos law is a principal driver of natural selection: A proposition on the evolution of recently emerged coronaviruses

**DOI:** 10.1371/journal.pone.0290453

**Published:** 2023-08-24

**Authors:** Pier Francesco Roggero, Arianna Calistri, Giorgio Palù

**Affiliations:** 1 Department of Molecular Medicine, University of Padua, via A. Gabelli, Padua, Italy; 2 Italian Medicines Agency, Via del Tritone, Rome, Italy; University of Bologna / Romagna Local Health Authority, ITALY

## Abstract

Here we propose that viruses emerging in the human population undergo an evolution that is conditioned by the rules of chaos. Our data support the notion that the initial growth rate “*r*” affects the chances of the virus to establish a long-lasting relationship with the new host. Indeed, an emerging virus is able to spread and adapt only when it displays an initial *r* falling in a range frankly associated with chaotic growth.

## Introduction

Members of the same species differ from each other by genetic and, therefore, phenotypic characteristics, i.e. morphological and functional features, the result of the interaction of the genotype with the environment. Darwin’s natural selection is a key evolutionary mechanism according to which, within the genetic diversity of populations, there is a progressive and cumulative increase of individuals optimized for their living environment [1]. This variability derives from random genetic mutations, in the sense that they occur by chance, during the generations [2]. Those mutations that confer advantages, e.g. in terms of survival and reproduction, are maintained and spread in the population. Genes heralding an adaptive advantage can be vertically transmitted to the progeny with a progressive affirmation of good genes at the expense of the less useful, non-useful or harmful ones.

Interestingly, it has been proposed that evolution tends towards states of greater disorder defined as entropy [3, 4]. The entropy of systems increases not because of an ominous tendency of the universe toward disorder, but due to a tendency toward most probable states, where most probable means those states corresponding to a greater number of molecular, atomic and subatomic variations. Chaos is therefore much more likely than the order.

Based on these considerations, the chaos inherent in the environment would represent the first motor of evolution leading to the emergence of mutations that will be selected according to the criterion of advantageousness. The species will progressively adapt to the environment until an equilibrium is reached, which is perturbed when a novel change arises, once again due to the intrinsic chaos of the environment. Chaos itself would be driven by an attractor entity presiding over evolution, thus not representing pure randomness [5, 6].

To support the proposition that chaos is the master of evolution, we adopted viruses as a model since they are the simplest biological entities most prone to mutate. In particular, we considered the recently emerged coronaviruses, i.e. the pandemic Severe Acute Respiratory Syndrome CoronaVirus-2 (SARS-CoV-2), the etiological agent of COVID-19 [7, 8], as well as the non-pandemic SARS-CoV-1 and Middle East Respiratory Syndrome (MERS)-CoV [9]. These viruses are particularly interesting to investigate and compare in terms of mutation, evolution and adaptation, since they possess the largest genome among riboviruses and share a common zoonotic origin. Moreover, all epidemiological parameters are available for these coronaviruses as they became human pathogens in recent years and were all classified as public health emergencies of international concern (PHEIC). An approach at the cellular level would not be feasible since it will be constrained by the cellular model utilized and inevitably fail to give a real picture of the whole phenomenon as it occurs *in vivo*. When these viruses replicate, random mutations occur that can be advantageous or unfavorable. If they are advantageous, increasing for example the ability of the virus to enter into target cells, or to evade the immune response, as the mutations closely located in the RBD region of the S protein of SARS-CoV-2 [10], the mutant virus will take over the wild type parental strain and this will eventually disappear. Mutations tend to converge in specific regions of the genome, as only favorable changes are maintained and transmitted to the progeny that guarantee viral survival. Indeed, due to their nature of obliged intracellular parasites viruses co-evolve with the host cells to adapt and persist as long as possible in their ecological niche. In conclusion, two conditions determine viral features: 1) the emergence of random mutations; 2) the time of co-evolution with the host. Mutations will be transmitted if they increase the probability of the virus to be maintained in the host population.

## Materials and methods

### Laboratory-confirmed cases of infections

In this study the number of laboratory-confirmed cases of different viral infections registered over time were adopted to calculate a set of parameters. This number was retrieved by consulting publically accessible repositories, that are reported below along with the last accession date.

SARS-CoV-2: https://www.worldometers.info/coronavirus/#main_table, last accessed on 06/07/2023

SARS-CoV-1: https://www.who.int/health-topics/severe-acute-respiratory-syndrome#tab=tab_1, last accessed on 09/02/2023

MERS-CoV: https://www.emro.who.int/health-topics/mers-cov/mers-outbreaks.html, last accessed on 09/02/2023

Ebola virus: https://apps.who.int/gho/data/view.ebola-sitrep.ebola-summary-20150331?lang=en (World Health Organization Report), last accessed on 09/02/2023

### Mathematical equations

In this study, the following mathematical equations were adopted.

**Chaos theory logistic map**:

$$x\;\left(n+1\right)=r*x\;\left(n\right)*\left[1-x\left(n\right)\right]$$ (1)

where:

[*x* (*n* + 1)] is the population size at any given time,

[*x*(*n*)] is the population size at the previous step;

*r* is the growth rate

**Growth rate**:

r=lnPtP0t (2)

where:

*P*_*t*_ = the total number of infections at time t

*P*_0_ = the initial number of infections

t = time of data collection

**Weighted arithmetic mean of r**:

$$\bar{r}=\frac{\sum_{i=1}^{n}w_{i}r_{i}}{\sum_{i=1}^{n}w_{i}}$$ (3)

where:

*r*_*i*_ is one of the *r* calculated for a specific virus in a certain time frame

*w*_*i*_ is the corresponding weight (i.e. the corresponding time of data collection analyzed)

**Nominal daily interest rate**:

$$P_{t}=P_{0}\;\times {\left(1+d\right)}^{t}$$ (4)

where,

*d* is the unknown nominal daily interest rate

t is the time of data collection

## Results and discussion

Our proposition here is that an initial chaotic growth enables an emerging virus to acquire mutations that facilitate its adaptation to the new host, resulting, in the absence of effective Public Health interventions and depending on the biological characteristics of the virus, in an endemic/epidemic infection.

**Fig 1:**
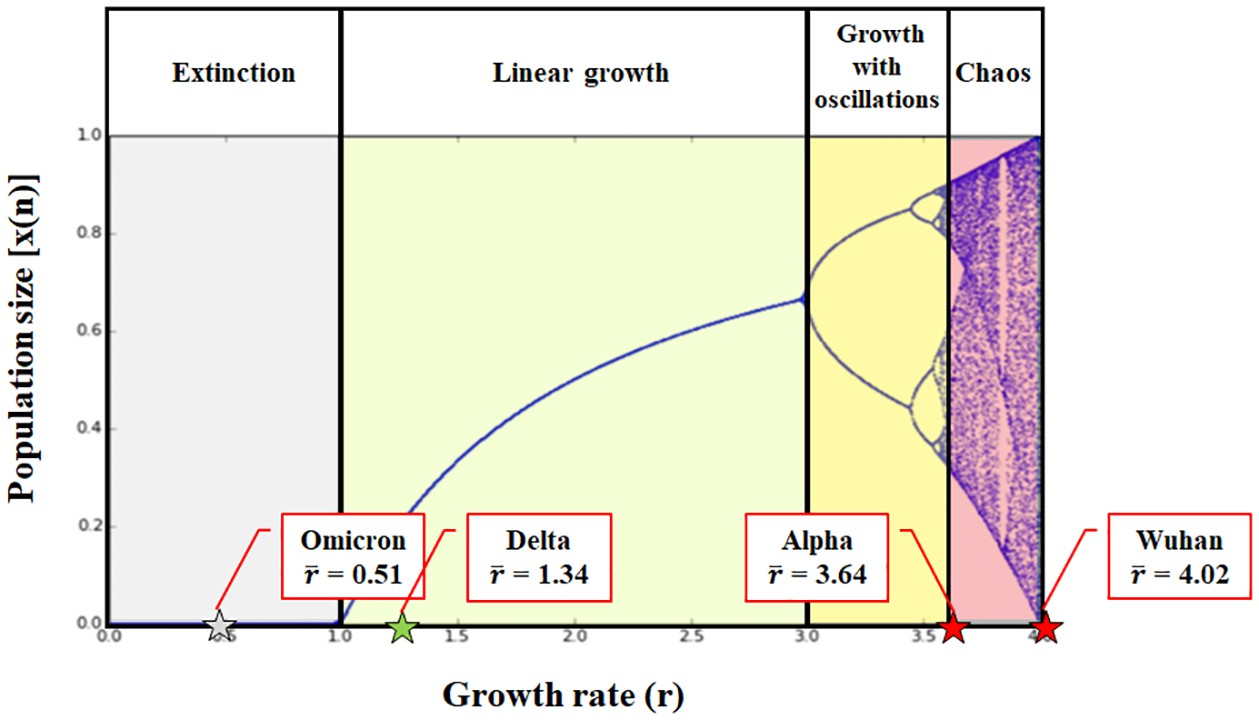
Graphical representation of the logistic map of chaos. The figure reports a classic graphic representation of the equation x (n+1) = r*x (n)*[1-x(n)] for 1 ≤ *r* ≤ 4. The graph is divided into 4 zones according to the predicted type of growth of the size population. In detail, the grey zone encompasses the r values leading to the population extinction; the light green zone is the one characterized by a linear growth; the yellow zone is the one depicting a growth with oscillations; the pink one represents the chaos zone. Stars point to the $\bar{r}$ values of SARS-CoV-2 variants calculated as explained in the text.

To prove this proposition, we took into account the chaos theory logistic map [11]:

$$x\;\left(n+1\right)=r*x\;\left(n\right)*\left[1-x\left(n\right)\right]$$

whose graphical representation, per 0 < *r* < 4, is depicted in Fig 1. The equation reflects the notion that, given a dynamic system and a wide range of starting conditions, the set of states toward which the system itself evolves after a sufficiently long period of time is defined attractor. When a system has a value close enough to the attractor and it is slightly disturbed, it tends to remain close to this value (5, 6). Talking about population, the attractor will be represented by the value reached by the population size. In this case, [*x*(*n* + 1)] will be the size population at any given time and it will be a function of the growth rate *r* and of the population size at the previous step [*x*(*n*)]. The parameter *r* reflects the combined effect of reproduction and density-dependent mortality (starvation). If *r* is too low, the population will extinguish. Higher *r* values can either results in a population size that settles to a stable value or fluctuates across a series of values. At certain *r* values the logistic map produces chaos. Interestingly, *x* (*n*) remains in the range [0,1] if *r* falls in the interval [0,4]. If r > 4 the sequence diverges for almost all the initial values and leaves the interval [0, 1], leading to negative population sizes. In more details, in the interval 0 < *r* < 4, for *r* < 1.0, while the model iterates the population size is attracted over time to 0 (extinction); for 1.0 < r < 3, the population size is attracted to stable and exact values; for 3 < r < 3.56995 (herein indicated as 3.57 for simplicity) the population size oscillates between a discrete and precise 2^n^ number of values (with n ≥ 1 and integer), depending from the actual r; at 3.57 < r < 4, the population grows in a chaotic way, jumping between all possible values. However, the produced randomness is only apparent, as the model is still driven by deterministic rules. In summary and as exemplified in Fig 1:

- 3.57 < *r* < 4 the population grows in chaotic way (pink part of the logistic map)
- 3 < *r* < 3.57 the population grows with oscillations in its size (yellow part of the logistic map)
- 1 < *r* < 3 results in a linear growth unaffected by chaos and the size will settle down to a specific value (light green part of the logistic map)
- *r* < 1 the population size goes to 0 leading to extinction (grey part of the logistic map)

First, by adopting data already in the public domain, we applied the model to SARS-CoV-2, the coronavirus most recently emerged in the human population (year 2020) and responsible for the current pandemic of COVID-19 [7, 8]. We used the growth of the number of infections worldwide and over time as an indicator of the virus ability to spread through the population, and, thus, of its fitness and adaptation to the new host. We calculated SARS-CoV-2 doubling period T, taking into account the time needed to go from 10 million infections to 20, then to 40, 80, 160, 320 and 640 (the infections reached 691 million on 06/07/2023). T was correlated to *r*, by exploiting the simple growth rate formula:

r=lnPtP0t

In this formula *P*_*t*_ represents the total number of infections at time t, *P*_0_ is the initial number of infections, while t is time frame of data collection taken into account. As a consequence in order to calculate T, i.e. the time expressed in days requested for doubling the initial number of infected individuals, *P*_*t*_ = 2 *P*_0_ and the formula becomes:

$$T\;=\frac{\text{ln}\;2\;\times \;365}{r}$$

or

$$r\;=\frac{\text{ln}\;2\;\times 365}{T}$$

The weighted arithmetic mean was also calculated by applying the formula:

$$\bar{r}=\frac{\sum_{i=1}^{n}w_{i}r_{i}}{\sum_{i=1}^{n}w_{i}}$$

where *r*_*i*_ is one of the *r* calculated for the virus in a certain time frame, while *w*_*i*_ is the corresponding weight expressed by the specific time frame of data collection under evaluation.

Finally, we determined the relative nominal daily interest rates (*d*) by adopting the formula:

$$P_{t}=P_{0}\;\times {\left(1+d\right)}^{t}$$

where t is again the analyzed time frame of data collection. If *t* = *T* the formula becomes

$$P_{T}\;=P_{0}\;\times {\left(1+d\right)}^{T}$$

The growth rate *r*, the corresponding $\bar{r}$ and *d* were calculated for the original Wuhan SARS-CoV-2, as well as for the Alpha, Delta and Omicron variants over the period of their dominance. As shown in Table 1, the parameter *r* decreases from a value close to 4 (*r* = 4.02, at the beginning of the pandemic caused by Wuhan SARS-CoV-2) to a weighted mean value of 0.51 (to date, Omicron variant). The overall findings indicate an initial chaotic growth, which lasted throughout the year 2020 with the Wuhan and the following uAlpha variant of SARS-CoV-2. Next, with the availability of vaccines, antivirals and monoclonal antibodies along with an increasing population immunity from natural infection, growth became linear and no longer chaotic. Of note, the last T value reported in Table 1 does not represent a doubling time but the situation of the last 239 days of data collection, and the relative *r*, as well *d*, are calculated taking into account this aspect. Importantly when the ratio between *r* and *d* expressed as percentage [*d* (%)] is calculated for each T, an almost constant value (*r* ≈ 3.6) is obtained demonstrating that the two parameters are directly proportional, as expected.

**Table 1:** Parameters for SARS-CoV-2.

START	END	T	SARS-CoV-2 variant	P	*d*	*d* (%)	*r*	$\bar{r}$	*r*/*d*(%)
22 JAN 2020	25 JUN 2020			10					
26 JUN 2020	07 AUG 2020	63	**Wuhan**	20	0.0111	1.11%	4.02	**4.02**	3.62
08 AUG 2020	15 OCT 2020	69	**Alpha**	40	0.0101	1.01%	3.67		3.63
16 OCT 2020	24 DEC 2020	70	**Alpha**	80	0.0099	0.99%	3.61	**3.64**	3.65
25 DEC 2020	10 MAY 2021	137	**Delta**	160	0.0051	0.51%	1.85		3.62
11 MAY 2021	05 JAN 2022	240	**Delta**	320	0.0029	0.29%	1.05	**1.34**	3.64
06 JAN 2022	09 NOV 2022	308	**Omicron**	640	0.0022	0.22%	0.82		3.65
10 NOV 2022	06 JUL 2023	239^a^	**Omicron**	691	0.00032	0.032%	0.117	**0.51**	3.66

The table reports all the relevant information and values adopted to calculate *d*, *r* and $\bar{r}$ for the pandemic SARS-CoV-2, using the equations described in the text. Used data were retrieved from https://www.worldometers.info/coronavirus/#main_table, last accessed on 06/07/2023. P represents the total number of cases after each T and reported numbers should be multiplied by 10^6^. T is expressed in days.

^a^ this time frame does not correspond to a doubling time. This aspect was taken into consideration for the calculation of all the relative parameters listed in the table.

Two additional coronaviruses emerged quite recently in the human population, i.e. SARS-CoV-1 and MERS-CoV (9). SARS-CoV-1 emerged in the year 2002 causing an epidemic characterized by a severe respiratory illness with a significant mortality rate in most of the confirmed cases [12]. The virus was eradicated from the human population by the year 2004. The calculated *r* values for SARS-CoV-1 are ≫ 4 from the beginning of the outbreak till May 2004 (Table 2). When the virus started to be efficiently contained by the Public Health response, the *r* parameter dropped to a value < 1 (*r* = 0.06) along with the weighted arithmetic mean (Table 2). It has to be mentioned that, as for SARS-CoV-2, this last *r* value does not derive from a doubling time, but it is still interesting to report as it covers the last year of virus circulation. The relevance of this value is supported by the respective *r*/*d*(%) that is ≈ 3.6, as expected.

**Table 2:** Parameters for SARS-CoV-1.

START	END	T	P	*d*	*d*(%)	*r*	$\bar{r}$	*r*/*d*(%)
16 NOV 2002	01 MAR 2003		1000					
02 MAR 2003	05 APR 2003	34	2000	0.0206	2.06	7.44		3.61
06 APR 2003	25 APR 2003	19	4000	0.0372	3.72	13.32		3.58
26 APR 2003	20 MAY 2003	24	8000	0.0293	2.93	10.54		3.6
21 MAY 2003	19 MAY 2004	364^a^	8500	0.00017	0.017	0.06	**1.77**	3.53

The table reports all the relevant information and values adopted to calculate *d*, *r* and $\bar{r}$ for SARS-CoV-1. Used data were retrieved from https://www.who.int/health-topics/severe-acute-respiratory-syndrome#tab=tab_1, last accessed on 09/02/2023. P represents the total number of cases after each T. T is expressed in days.

^a^ This time frame does not correspond to a doubling time. This aspect was taken into consideration for the calculation of all the relative parameters listed in the table.

By contrast, MERS-CoV, a coronavirus that originated in Saudi Arabia in 2012 [13], shows low *r* values since the beginning of the epidemic, with an overall weighted arithmetic mean < 1, i.e. 0.39 (Table 3). In this case, the available data did not allow to determine the exact doubling time T. Instead, the number of days (*t*) requested to reach a certain total P value is reported and adopted to calculate *r*. The relevance of the obtained results is supported by the *r*/*d*(%) values that are again constant and ≈ 3.6.

**Table 3:** Parameters for MERS-CoV.

START	END	*t*	P	*d*	*d* (%)	*r*	$\bar{r}$	*r*/*d*(%)
01 JUN 2012	31 DEC 2012	213	14					
01 JAN 2013	31 DEC 2013	365	114	0.0057	0.57	2.10		3.68
01 JAN 2014	31 DEC 2014	365	495	0.004	0.4	1.47		3.67
01 JAN 2015	31 DEC 2015	365	987	0.0019	0.19	0.69		3.63
01 JAN 2016	31 DEC 2016	365	1236	0.0006	0.06	0.22		3.75
01 JAN 2017	31 DEC 2017	365	1486	0.0005	0.05	0.18		3.68
01 JAN 2018	31 DEC 2018	365	1633	0.0003	0.03	0.09		3.14
01 JAN 2019	31 DEC 2019	365	1845	0.0003	0.03	0.12		4.07
01 JAN 2020	01 MAY 2023	1216	2604	0.0028	0.028	0.10	0.39	3.69

The table reports all the relevant information and values adopted to calculate *d*, *r* and $\bar{r}$ for MERS-CoV. Data from https://www.emro.who.int/health-topics/mers-cov/mers-outbreaks.html, last accessed on 09/02/2023. P represents the number of cases registered after each time of data collection. The number of days (t) requested to reach a certain total P value is reported and adopted to calculate r.

Interestingly, *r* is always < 3.57, even in the first two years of virus circulation, becoming < 1 since 2015. This coronavirus, that caused a severe respiratory syndrome with a high lethality rate, has affected roughly 3000 people (2604 laboratory-confirmed cases) since its emergence in the human population and it is still present in some areas of the Middle and Far East [13]. MERS-CoV, however, has its natural host reservoir in dromedary camels and it is hardly transmitted from man to man [14]. Importantly, at variance with SARS-CoV-2, neither SARS-CoV-1 (*r* ≫ 4 at the onset) nor MERS-CoV (*r* < 3.57 at the onset and rapidly going below 1) became pandemic.

Finally, as an internal control of the proposition, we applied this model to a different RNA virus, for which epidemiological information are as well available, i.e. the negative single-stranded RNA Ebolavirus. Although this virus occasionally appeared in the human population, it was so far unable to become endemic or pandemic. We took into account the West African Ebola virus epidemic that was the largest registered so far. During this epidemic, Ebola virus was initially present in Guinea and rapidly spread to the neighboring Countries of Liberia and Sierra Leone with smaller outbreaks occurring in Senegal, Nigeria and Mali [15]. Data reported in Table 4 indicate high values of *r* since the beginning of the epidemic, as in the case of SARS-CoV-1 outbreak.

**Table 4:** Parameters for Ebola virus.

START	END	T	P	*d*	*d* (%)	*r*	$\bar{r}$	*r*/*d*(%)
01 DEC 2013	14 JUL 2014		1000					
15 JUL 2014	09 AUG 2014	25	2000	0.0281	2.81	10.12		3.60
10 AUG 2014	31 AUG 2014	21	4000	0.0336	3.36	12.05		3.59
01 SEP 2014	05 OCT 2014	34	8000	0.206	2.06	7.44		3.61
06 OCT 2014	22 OCT 2014	16	16000	0.0443	4.43	15.81		3.57
23 OCT 2014	13 APR 2016	538^a^	28700	0.00109	0.11	0.40	1.94	3.64

The table reports all the relevant information and values adopted to calculate *d*, *r* and $\bar{r}$ for Ebola virus. Used data were retrieved from “Ebola data and statistics” (World Health Organization Report), last accessed on 09/02/2023. P represents the total number of cases after each T. T is expressed in days.

^a^ This time frame does not correspond to a doubling time. This aspect is taken into consideration for the calculation of all the respective parameters listed in the table.

Notably, also in the case of this virus, *r is* ≫ 4 at the onset and throughout the first 11 months of circulation. Next, the *r* value drops (*r* < 1 and = 0.40), likely due to a combination of Public Health, therapeutic and prophylactic interventions, along with the likely inability of the virus to adapt to the human population. Although this last value does not derive from a doubling time, it represents the parameter calculated for the last 538 days of viral circulation, and, thus, it can be considered highly relevant. In agreement, the respective *r*/*d*(%) value is ≈ 3.6.

## Conclusions

Overall our findings suggest that the *r* value characterizing a virus when it emerges in the human population (initial *r*) is likely reflecting its intrinsic biological characteristics that affect its ability to spread and its chances to establish a long-lasting relationship with the new host. Indeed, the probability for an emerging virus to become pandemic or endemic appears to be low if its initial *r* value is ≫ 4 (SARS-CoV-1 and Ebolavirus). This conclusion is mathematically supported, since, as already mentioned, the population sizes can assume negative values for *r* > 4. On the other hand, a virus, when accidentally transmitted to man, seems not prone to evolve further also if its initial *r* is < 4 but it does not fall within the range of values frankly associated with chaotic growth (i.e. 3.57 < *r* < 4), This would be the case of MERS-CoV (initial *r* = 2.10). By contrast, when an emerging virus displays an *r* value falling in the [3.57,4] interval, a chaotic growth occurs, which enables virus evolution towards its adaptation to the new host. This is indeed the case only for SARS-CoV-2 whose initial *r* is very close to 4, being 4.02. SARS-CoV-2 chaotic growth is replaced over time by a linear growth, allowing virus establishment in the human population. Currently, the calculated *r* is < 1, a value mathematically associated to the extinction.

In conclusion, we propose that viruses arising in a new host evolve according to the theory of chaos. In the case of viruses able to adapt to the new host (SARS-CoV-2, initial *r* = 4.02), an initial chaotic growth will favor genome mutations and drive virus evolution towards its establishment in the population. On the other hand, viruses that will not reach an equilibrium with the new host (SARS-CoV-1 and Ebola virus) display initial high *r* values (*r* = 7.44 and *r* = 10.12, respectively), fail to stably circulate in the human population and are either naturally eradicated or eradicated from the host population by effective containment measures. Finally, emerging viruses that start with an *r* value < 3.57 (MERS-CoV) are not likely to become pandemic, as they are not prone to mutate and adapt to the new host.

Although the chaos theory was applied here directly to viral spread in the population, it is tempting to speculate that the same rules could master the emergence of mutations within the viral genome. In this context, favorable mutations would be those that are spatially close enough to each other within specific regions (attractors), as they can guarantee virus survival. In other words, in emerging viruses prone to establish a long-lasting relationship with the new host, the genome would mutate following attractor elements that inherently drive this evolution towards the most favorable state to allow virus survival and fitness. This process obeys to Darwin’s natural selection; mutations, which would then be defined “chaotic” instead that “random”, would arise through predetermined hierarchic imprinting that is working in topically clustered genomic hot spots, as it is indeed the case of the above-mentioned gene coding for SARS-CoV-2 S protein [10].

We are well aware that the proposition we have advanced here is based on a simplification of very complex interplays. However, we still believe that the *r* parameter, especially the initial one, viewed in the context of virus biology and Public Health, could provide useful information about the possibility that an emerging virus could adapt to the human species, becoming a threat to humankind.

Chaos has been recently proposed as a general law that determines how physical, chemical and biological events take place in the universe in a fashion that seems opposed to that of chance or randomness. In the realm of viruses, we assume that the cause are the initial biological characteristics (e.g. virus genetic setup) while the effect is the chaotic process of virus adaptation to the newly parasitized host. Thus, chaos would also be presiding over the most elementary biological entities.
